# A Review Paper about Deep Learning for Medical Image Analysis

**DOI:** 10.1155/2023/7091301

**Published:** 2023-05-29

**Authors:** Bagher Sistaninejhad, Habib Rasi, Parisa Nayeri

**Affiliations:** ^1^Seraj Institute of Higher Education, East Azerbaijan, Tabriz, Iran; ^2^Sahand University of Technology, East Azerbaijan, New City of Sahand, Iran; ^3^Khoy University of Medical Sciences, West Azerbaijan, Khoy, Iran

## Abstract

Medical imaging refers to the process of obtaining images of internal organs for therapeutic purposes such as discovering or studying diseases. The primary objective of medical image analysis is to improve the efficacy of clinical research and treatment options. Deep learning has revamped medical image analysis, yielding excellent results in image processing tasks such as registration, segmentation, feature extraction, and classification. The prime motivations for this are the availability of computational resources and the resurgence of deep convolutional neural networks. Deep learning techniques are good at observing hidden patterns in images and supporting clinicians in achieving diagnostic perfection. It has proven to be the most effective method for organ segmentation, cancer detection, disease categorization, and computer-assisted diagnosis. Many deep learning approaches have been published to analyze medical images for various diagnostic purposes. In this paper, we review the work exploiting current state-of-the-art deep learning approaches in medical image processing. We begin the survey by providing a synopsis of research works in medical imaging based on convolutional neural networks. Second, we discuss popular pretrained models and general adversarial networks that aid in improving convolutional networks' performance. Finally, to ease direct evaluation, we compile the performance metrics of deep learning models focusing on COVID-19 detection and child bone age prediction.

## 1. Introduction

Computer-aided diagnosis (CAD) has emerged as one of the most important research fields in medical imaging. In CAD, machine learning algorithms are often utilized to examine the imaging data from historical samples of patients and construct a model to assess the patient's condition [1]. The developed model assists clinicians in making quick decisions. The most common imaging modalities used in medical applications are X-ray, computed tomography (CT), magnetic resonance imaging (MRI), positron emission tomography (PET), and ultrasound. The sole aim of medical image processing would be to improve the interpretability of the information illustrated [2]. The following are the main categories of medical image analysis: enhancement, registration, segmentation, classification, localization, and detection [3].

Earlier, medical images were processed using low-level methods, such as thresholding, region growing, and edge tracing [4]. Meanwhile, the growth in size and scope of medical imaging data has fueled the evolution of machine learning techniques in medical image analysis. However, since such methods rely on handcrafted features, algorithm design requires manual effort. These constraints of conventional machine learning approaches have risen to the notion of artificial neural networks (ANNs). Factors such as data availability and computational processing capabilities facilitate the deepening of ANNs [5]. The emergence of deep learning techniques like convolutional neural networks has widened the possibilities for the automation of medical image processing.

A convolutional neural network (CNN) is a class of neural networks meant to handle pixel values. CNN makes image classification more scalable by employing linear mathematical concepts to detect patterns inside an image. While traditional CNN architectures consisted solely of convolutional layers placed on top of one another, modern architectures such as Inception, ResNet, and DenseNet come up with new and innovative approaches to building convolutional layers in a way that makes learning more efficient [6].

CNN can be employed as a feature extractor as well. Feature extraction aims to convert raw pixel data into numerical features that can be processed while keeping the information in the original data set. Traditional feature extractors can be replaced with CNNs, which can extract complex features that express the image in much more detail. The resulting features are then fed into a classifier network or used by typical machine learning algorithms for classification [7].

Despite the fact that deep CNN architectures exhibit cutting-edge performance on computer vision problems, there are some concerns about using CNN in the radiology field. In 2014, Goodfellow et al. discovered that introducing a little bit of noise to the original information can readily deceive neural networks into misclassifying items [8]. Furthermore, since the efficiency of deep learning is often based on the volume of input data, CNN requires large-scale, well-annotated radiology images. Building such databases in the medical industry, on the other hand, is costly and labor-intensive.

In this study, we summarize the current developments in deep learning approaches for medical image analysis. The paper is organized as follows: first, survey papers related to medical image analysis are discussed in Section 2. Then, in Section 3, CNN models employed in the radiology field and approaches for improving CNN performance are described. Following that, the finding of models aimed at detecting COVID-19 and predicting child bone age are reviewed in Section 4. And finally, the conclusion is set out.

## 2. Related Works

This section discusses the survey papers on medical image analysis using deep learning-based algorithms. Hu et al. [9] described four deep learning architectures used for image analysis: CNN, fully convolutional networks (FCN), deep belief networks, and autoencoders. They also compiled recent works on cancer identification and diagnosis.

Liu et al. [10] concentrated on deep learning-based medical image segmentation. They began by explaining the deep learning framework deployed to segment medical images. Then, state-of-the-art segmentation architectures such as FCN, U-Net, and generative adversarial network (GAN) were examined.

Shin et al. [11] first studied two medical diagnostic problems, namely, interstitial lung disease detection and thoracoabdominal lymph node classification, using three CNN models: AlexNet, CifarNet, and GoogLeNet. Then looked at how transfer learning enhanced the performance of each model.

Kazeminia et al. [12] provided a broad insight into the current studies on GANs for medical applications, discussed the limitations and opportunities of the existing techniques, and elaborated on potential future work.

Fu et al. [13] highlighted the current advancements in medical image segmentation by deep learning. They divided the approaches reviewed into two main groups: pixel-by-pixel classification and end-to-end segmentation, and discussed the performance, limits, and future potential of each group.

## 3. Medical Image Analysis Using Deep Learning

The primary focus of medical image analysis is to find out which regions of the anatomy are affected by the disease to aid physicians in learning about lesion progression. The analysis of a medical image is mostly reliant on four steps: (1) image preprocessing, (2) segmentation, (3) feature extraction, and (4) pattern identification or classification [14]. Preprocessing is used to remove unwanted distortions from images or improve image information for further processing. Segmentation refers to the process of isolating regions, such as tumors, and organs, for further study. The process of extracting precise details from the regions of interest (ROIs) that aid in their recognition is known as feature extraction. Based on extracted features, classification assists in categorizing the ROI.

We have compiled a list of research papers primarily concerned with segmentation and classification in medical imaging. Following the review of CNN, we have outlined some techniques for improving CNN's performance.

### 3.1. Convolutional Neural Network

A CNN is a supervised deep learning framework that can accept images as input, allocate filters to convert image pixels into features, and apply those features to distinguish one data from another. It is generally composed of three layers: the convolutional layer, pooling layer, and fully connected layer. The convolutional layer is the initial layer of a convolutional network. After that, more convolutional layers or pooling layers can be added, with the fully connected layer being the last.

The convolutional block draws the features from the image, from which the network can analyze and obtain hidden correlations. Pooling layers are applied to reduce the size of the convolved features, referred to as downsampling. Fully-connected layers execute classification tasks depending on the features retrieved by the preceding layers. While convolutional layers generally adopt rectified linear unit (ReLu) function to activate neurons, Fully connected layers employ a softmax activation function or traditional machine learning classifiers (SVM, KNN, etc.) to classify inputs.

### 3.2. Overview of Works

Despite the deep network's ability to extract features with more precision, it requires a lot of computing resources. Therefore, Badža and Barjaktarović [15] introduced a simple CNN model with two convolutional blocks for classifying brain tumors using MRI images. While evaluating 3064 MRI images, the model attained the best accuracy of 95.56% using 10-fold cross-validation. Rachapudi and Lavanya presented an efficient CNN architecture with a 22.7% error rate to classify the colorectal cancer histopathological images. To prevent overfitting, the model included five convolutional blocks, each containing a dropout layer [16].

The deep learning architecture for image segmentation comprises an encoder and a decoder. The encoder uses filters to extract features from the image, whereas the decoder is in charge of producing the final output, often a segmentation mask containing the object's shape. A fully convolutional network (FCN) is an encoder-decoder model that lacks dense layers in favor of 1 × 1 convolutions to serve the function of fully connected layers [17]. Sun et al. developed a 3D FCNN-based model for multimodal brain tumor image segmentation. The encoder had four pathways for extracting multiscale image features [18]. Then, these four feature maps were fused and fed to the decoder. By experimental validation on the brain tumor segmentation challenge dataset 2019 (BraTS2019), the model segmented the dataset with Dice.

Similarity Coefficient metrics (DSC) of 0.89, 0.78, and 0.76 for the complete, core, and enhanced tumor, respectively. In 2015, Ronneberger et al. introduced U-Net to deal with biomedical image segmentation that can learn from a small number of annotated medical images [19]. U-Net is a U-shaped encoder-decoder-based framework consisting of four encoder and four decoder blocks connected by skip connections. Dharwadkar and Savvashe employed U-Net architecture to design a ventricle segmentation model for heart MRI images. There are four layers in the original U-Net, but only three layers were employed in this model [20]. For the right ventricle segmentation challenge (RVSC) dataset, the proposed model obtained a dice score of 0.91.

For segmenting the left ventricle from cardiac CT angiography, Li et al. introduced U-Net with 8 layers. The exhibited U-Net model comprised eight encoder and eight decoder blocks. To further improve the network's efficiency, residual blocks in the form of skip connections were introduced into each encoder and decoder block [21]. The model was trained using 1600 CT images from 100 patients, resulting in a DSC of 0.9270 ± 139. Li et al. [22] introduced an attention mechanism between nested encoder-decoder paths in the U-Net++ [23] architecture to improve the understanding of the study area in liver segmentation. The model achieved a DSC of 98.15% through the experimental analysis of the liver tumor segmentation challenge dataset 2017 (LiTS2017).

V-Net extends U-Net by processing 3D MRI images with 3D convolutions [24]. Guan et al. developed a V-Net-based framework for separating brain tumors from 3D MRI brain images. In the developed framework, the squeeze and excite (SE) module and attention guide filter (AG) module were integrated into V-Net architecture to suppress irrelevant information and enhance segmentation accuracy [25]. When tested on the BraTS2020 dataset, the model obtained dice metrics of 0.68, 0.85, and 0.70 for the complete, core, and enhanced tumor, respectively.

Mask regional CNN is another CNN variant used in medical image segmentation. Mask R-CNN is a two-phase object identification and segmentation architecture. The first stage, known as the region proposal network (RPN), returns potential bounding boxes, whereas the second stage generates the segmentation mask from each box [26]. Dogan et al. introduced a hybrid model combining U-Net and mask R-CNN for pancreas segmentation from CT images. The proposed system was composed of two parts: pancreas detection and pancreas segmentation. In pancreas localization, the region proposal network, in conjunction with the mask production network, was used to determine the bounding boxes of the pancreas portion, and the subregion centered by the rough pancreas region was sliced [27]. Finally, the cropped subregion was sent to U-Net for precise segmentation. The average DSC for the two-phase approach demonstrated on the 82 abdominal CT scans was 86.15%.

### 3.3. Improving the Performance of CNN

The CNN model is often used for image classification because it achieves better accuracy with a low error rate. However, it needs large datasets to generalize the hidden correlations found in the learning data. Here, we have discussed two approaches that may optimize the performance of CNN: (1) transfer learning and (2) general adversarial network (GAN).

### 3.4. Transfer Learning

Transfer learning is an effective strategy to train a network with a limited dataset. Here, the model is pretrained using a large-scale dataset, like ImageNet having 1.4 million images divided into 1000 categories and then applied to the problem at hand [28]. The major pretrained CNN architectures for image classification are as follows:

*LeNet-5*: LeNet-5 [29], a 7-level convolutional network presented by Lecun et al. in 1998, was the first of its kind. The model was designed to classify handwritten digits and tested on the MNIST standard dataset, with a classification accuracy of roughly 99.2%

*AlexNet*: the network's design was quite similar to LeNet, but it was deeper, with more filters per layer. It contains five convolution layers and three fully-connected layers. To control overfitting, it employs a dropout mechanism in fully connected layers [30]

*Visual Geometry Group at Oxford (VGGNet)*: VGGNet typically consists of 16 layers with a lot of 3 × 3 filters of stride one [31]. It is now the most popular method for extracting features from images. VGGNet, on the other hand, has 138 million parameters, which are difficult to manage

*InceptionV1/GoogLeNet*: the inception/GoogleNet architecture, presented by Christian Szegedy et al., has 22 layers. The Inception block does 1 × 1, 3 × 3, 5 × 5 convolutions, and 3 × 3 pooling at the input, and the outputs of these are stacked to send to the next inception module [32]. By using 1 × 1 convolutions in each module, GoogleNet can reduce the size of parameters to 4 million compared to AlexNet's 60 million

*Residual network or ResNet*: a residual network, often known as ResNet, is a 152-layer model. This network employs a VGG-19-inspired network design, with grouped convolutional layers followed by no pooling in between and an average pooling before the fully connected output layer [33]. The design is converted into a residual network by adding shortcut connections. This sort of skip connection has the advantage of training deep networks without problems caused by vanishing gradients

In image classification, either the pretrained model can be used as-is or modified for a given problem. The fine-tuning of a model can be accomplished using one of the following strategies: (1) train some layers while leaving the others frozen and (2) freeze the convolutional base only.

### 3.5. Overview of Works

The surveyed works on transfer learning are listed in Table 1. LeNet is a popular CNN model because of its simple architecture and shorter training time. Deep neural network models use the concept of the maxpooling layer to extract the most relevant features from a region. However, in medical image analysis, where quality is poor, pixels with lower intensities may hold critical information. Hence, Hazarika et al. introduced the minimum pooling layer in LeNet for Alzheimer's disease (AD) classification. In the modified LeNet [34], the min-pooling and max-pooling layers were merged, and the resulting layer replaced all maxpooling layers. According to the experimental study on 2000 brain images, the original LeNet model classified AD with 80% accuracy, while the revised LeNet attained an accuracy of 96.64%.

Hosny et al. introduced a fine-tuned AlexNet model to categorize skin lesions into seven classes using skin images. In the proposed architecture, the last three layers were replaced by new layers to make them suitable for classifying seven types of skin lesions [35]. The parameters of these new layers were initially set at random and then modified during the training. After training on 10,015 images, the model achieved an accuracy of 98.70% and a sensitivity of 95.60%. Dulf et al. trained and assessed five different models, including GoogleNet, AlexNet, VGG16, VGG19, and InceptionV3, to determine the best model for classifying the eight categories of colorectal polyps. The main criteria for adopting the network were sensitivity and F1-score [36]. Hence, InceptionV3 was chosen with an F1-score of 98.14% and a sensitivity of 98.13%. In InceptionV3 [37], the 5 × 5 convolutional layer is replaced with two 3 × 3 convolutional layers to lower the computational cost.

Hameed et al. demonstrated an ensemble deep learning strategy to categorize breast cancer into carcinoma and noncarcinoma using histopathology images. In this case, VGG models, namely VGG16 and VGG19, were used to design the framework. VGG19 has the same basic architecture as VGG16 with three additional convolutional layers. Besides the first block, the remaining four blocks were updated during training to fine-tune the models [38]. Finally, the tuned VGG16 and VGG19 models were ensembled, resulting in an overall accuracy of 95.29%.

Togacar et al. used both VGG16 and AlexNet to extract features for brain tumor classification from MRI images, where each model captured 1000 features [39]. Then, using the recursive feature elimination (RFE) feature selection algorithm, the obtained features were evaluated to identify the most efficient features. Finally, the SVM classifier gave 96.77% accuracy with 200 chosen features. Eid and Elawady presented a ResNet-based SVM for pneumonia detection using X-rays. The developed model preferred ResNet to get features from chest X-rays, then used a boosting algorithm to choose the relevant features and an SVM classifier to detect pneumonia based on those features [40]. The model had 98.13% accuracy after being trained on 5,863 X-rays.

Xiao et al. used a Res2Net-based 3D-UNet to segment the left ventricle from echocardiography images. To extract 3D features at multiple scales, the basic residual unit in Res2Net was replaced with a set of 3 × 3 × 3 filters [41]. Finally, a group of 1 × 1 × 1 filters merged feature maps from all groups. According to an experimental analysis of 1186 lung images from the Lung Nodule Analysis dataset 2016 (LUNA16), the model acquired a DSC of 95.30%. Goyal et al. used the mask R-CNN for segmenting kidneys from the MRI images. In the proposed work, InceptionResNetV2 was adopted as the CNN network to segment the kidneys. Then, to refine the segmentation result, postprocessing procedures such as eliminating any voxel that was not associated with the kidney and fill operation were performed [42]. The proposed model got a mean dice score of 0.904 after being evaluated with 100 scans.

### 3.6. Generative Adversarial Network

Goodfellow et al. introduced the generative adversarial network (GAN), a type of neural network meant for unsupervised learning. GANs generally are of two competing neural network models: a generator that creates new data samples that mimic training data and a discriminator that differentiates training data from the generator's output [43].

### 3.7. Overview of Works

GAN-based methods used in medical image analysis are listed in Table 2. Cirillo et al. introduced a 3D GAN to segment brain tumors using MRI images from the BraTS2020 dataset. The U-Net architecture-based generator resulted in the segmented tumor region. The GAN discriminator was given a 3D MRI image and its segmentation output from the generator as input and generated a precise segmentation mask [44]. The GAN model segmented the whole, the core, and the enhanced tumor with average dice scores of 87.20%, 81.14%, and 78.67%, respectively. Wang et al. developed a U-Net segmentation network and a discriminant network with multiscale feature extraction to enhance prostate segmentation accuracy [45]. The approach obtained a DSC value of 91.66% by demonstrating it on 220 MRI images.

Wei et al. used a combination of GAN and Masks R-CNN to segment the liver from CT images. In the improved mask R-CNN, the k-means algorithm was utilized to adjust the bounding box parameters using a Euclidean distance [46]. The GAN-based approach yielded an average DSC of 95.3% while evaluating 378 CT images. A V-Net and Wasserstein GAN-based model was explored by Ma et al. [47] to improve the efficiency of liver segmentation. The WGAN [48] model includes Wasserstein distance to fix the issue of GAN training instability. On two abdominal CT scan datasets, LiTS and CHAOS, the method achieved DSC of 92% and 90%, respectively. Zhang et al. proposed dense GAN coupled with the U-Net to separate lung lesions from COVID-19 CT images. A dense block with five layers [49] was introduced into the discriminator network to make the model more compact. The proposed model got a mean dice score of 0.683 when tested on 100 lung CT images.

GAN can also be used for data augmentation [65] (i.e., creating plausible examples to add to a dataset) to boost classifier accuracy. GAN was also used [66] to generate realistic skin cancer images. The generator generated high-quality training data, and the discriminator tried to distinguish the original data from the generator's data. Ahmad et al. developed an auxiliary GAN framework to assess the accuracy of skin cancer categorization. First, the variational autoencoder network was trained to obtain the latent noise vector, and the generator produced skin lesion samples from this informative noise vector [67]. The GAN used here not only decided whether the image was original or not but also predicted the image's class label with 92.5% accuracy.

## 4. Discussion

To make straightforward comparisons, we have summarized the outcomes of the papers based on COVID-19 identification and child bone age prediction.

### 4.1. COVID-19 Detection

COVID-Net, an open-access initiative, was launched in March 2020 to assist healthcare professionals in combating Corona Virus Disease 2019 (COVID-19) by leveraging the advancement of machine learning. Furthermore, it regularly releases deep learning models and benchmark datasets to keep up with the pandemic [79]. In response to this initiative, Wang et al. introduced COVID-Net, a deep CNN for COVID-19 identification from chest X-rays.

In the COVID-Net model, residual projection-expansion-projection-extension (PEPX) blocks which comprise four 1 × 1 convolutions, were introduced to enhance the efficiency of features while ensuring computational efficiency [80]. The model efficacy was verified using 13,975 X-ray images from the COVIDx dataset. According to the experimental findings, the model attained a precision of 98.9% and a recall of 91.0% to detect covid-19. The COVIDx is a large-scale dataset of chest X-ray images compiled from publicly available data sources. As of now, COVIDx consists of 30,882 X-ray images from 17,026 patients.

Table 3 shows the deep learning models that were employed for COVID-19 detection. We can observe from the methods studied that the classification accuracy is affected not only by the CNN model chosen but also by the size of the dataset, the type of modality, the data augmentation techniques, and the features opted for processing.

### 4.2. RSNA Pediatric Bone Age Challenge 2017

In 2017, the Radiological Society of North America (RSNA) held a contest to predict the children's bone age from the hand X-rays. The main goal of this challenge was to encourage people to develop machine learning models that could accurately estimate bone age from pediatric hand X-rays. The performance measure was the mean absolute error in months, the average absolute difference between predicted results and ground-truth bone age [81]. The bone age dataset [82], consisting of 14,236 left-hand X-ray images, was divided into a training set, a validation set, and a test set of 12611, 1425, and 200, respectively.

Table 4 summarizes some of the recent CNN-based methods that used the RSNA bone age benchmark dataset. The approaches stated were divided into two phases. The CNN model was used in the initial stage to carve up the hand region from the X-ray images. The second phase included a pre-trained model for extracting inherent features from the hand region and a regression layer to estimate bone age.

## 5. Conclusion

We have presented a detailed overview of newly published deep learning-based methods from 2019 to 2022 in medical imaging. Recent advances in deep learning architectures have the ability to boost diagnostic precision in medical imaging. On the other hand, deep learning necessitates a large volume of data to outperform traditional machine learning models. In practice, however, obtaining such datasets containing medical images is difficult. Transfer learning via pretrained models can help to solve this problem. There is a clear tendency toward modifying pretrained models to make them more appropriate for a specific task. This popularity is because pretrained models expedite training while ensuring good classification accuracy. Another trend is to employ GAN to enhance segmentation accuracy due to its capacity to generate high-quality medical images and imitate input data distribution. The GAN-based approaches have proven to be effective in resolving discrepancies between ground truth and model-generated segmentation masks. Also, GAN's ability to synthesize data can help solve difficulties such as lack of medical images or imbalanced data distribution, resulting in improved classification model performance.

## Figures and Tables

**Table 1:** Overview of pretrained models.

Ref.	Year	Model	Findings	Modality	Accuracy
[50]	2021	VGG16	Brain tumor classification	MRI	95.71%
[51]	2020	ResNet50	Brain tumor classification	MRI	97.2%
[52]	2020	GoogleNet	Alzheimer's disease classification	MRI	97.15%
[53]	2020	Ensemble of AlexNet, DenseNet121, ResNet18, GoogleNet, InceptionV3	Pneumonia detection	X-ray	96.4%
[54]	2020	AlexNet	Lung nodule classification	CT and X-ray	99.6%
[55]	2020	ResNet50	Breast tumor classification	Mammogram	85.71%
[56]	2020	ResNet50	Breast tumor classification	Histopathological images	99%
[57]	2021	VGG16	Breast tumor classification	Mammogram	98.96%
[58]	2020	DenseNet201	Skin lesion classification	Skin images	96.18%
[59]	2020	GoogleNet	Skin image classification	Skin images	99.29%
[60]	2021	VGG19	Thyroid nodule cell classification	Cytology images	93.05%
[61]	2021	GoogleNet	Thyroid nodule classification	Ultrasound	96.04%
[36]	2021	GoogleNet	Colorectal polyps classification	Gastrointestinal polyp images	98.44%
[62]	2020	Faster R-CNN+VGG16	Brain tumor segmentation and classification	MRI	77.60%
[63]	2021	U-Net+InceptionV3	Breast tumor segmentation and classification	Mammogram	98.87%
[64]	2020	Mask R-CNN+ResNet-50	White blood cells detection and classification	Cytological images	95.3%

**Table 2:** Overview of GAN-based methods.

Ref.	Approach	Findings	Metrics
[68]	Capsule network GAN+LeNet	Prostate image classification	Accuracy 89.20%
[69]	GAN+AlexNet	Parkinson's disease	Accuracy 89.23%
[70]	GAN+DenseNet121	Skin lesion classification	Accuracy 94.25%
[71]	GAN+InceptionV3	Breast mass classification	Accuracy 90.41%
[72]	GAN+ResNet50	Brain tumor classification	Accuracy 96.25%
[73]	3D U-Net, VGG16	Brain tumor segmentation	DSC 90.1%
[74]	U-Net, fully connected CNN	Breast tumor segmentation	DSC 88.41%
[75]	DeepLapV2 [76], FCN	Left ventricle segmentation	DSC 88.0%
[77]	U-Net, FCN	Whole heart segmentation	DSC 86.32%
[78]	Autoencoder, CNN	Lung lesion segmentation	DSC 62.0%

**Table 3:** Deep learning networks for COVID-19 identification.

Ref.	Deep learning model	Modality	Total samples			Evaluation metrics
			Normal	Pneumonia	COVID-19	
[80]	COVID-Net	X-ray	8,066	5,521	183	Accuracy 94.3%, precision 90.9%, recall 96.8%
[83]	EfficientNet	X-ray	8,066	5,521	183	Accuracy 93.9%, precision 100%, recall 96.8%
[84]	NASNet	X-ray	533	515	108	Accuracy 95%, precision 95%, recall 90%
[85]	GAN and VGG16	X-ray	721	0	403	Accuracy 95%, recall 90%
[86]	DenseNet103, ResNet18	X-ray	191	20	180	Accuracy 88.9%, precision 83.4%, recall 85.9%
[87]	ResNet101, ResNet152	X-ray	8851	9576	140	Accuracy 96.1%
[88]	DenseNet and graph attention network	X-ray	10192	7399	399	Accuracy 94.1%, precision 94.47%, recall 91.9%
[89]	VGG19	X-ray	3181	0	2049	Accuracy 98.36%
[90]	ResNet34, HRNet	X-ray	400	0	400	Accuracy 99.99%, precision 100%, recall 99.9%
[91]	VGG16	CT	49800	23652	80800	Accuracy93.57%, precision 89.40%, recall 94%
[92]	Deep long short-term memory network	CT	547	631	612	Accuracy 97.93%, recall 98.18%
[93]	VGG19	Ultrasound	235	277	399	Accuracy 100%

**Table 4:** CNN frameworks using RSNA bone age dataset.

Ref.	Segmentation	Regression	Mean absolute error (in months)
[94]	U-Net	VGG16	8.08
[95]	U-Net	Inception- ResNetV2	12.7744
[96]	DeepLabV3	MobileNetV1	8.200
[97]	U-Net	VGG16	9.997
[98]	CNN	MobileNetV3	6.2
[99]	Mask R-CNN	VGG19	6.38

## Data Availability

The data that support the findings of this study are openly available in with in the reference list.

## References

[1] Chan H. P., Hadjiiski L. M., Samala R. K. (2020). Computer-aided diagnosis in the era of deep learning. *Medical Physics*.

[2] Ritter F., Boskamp T., Homeyer A. (2011). Medical image analysis. *IEEE Pulse*.

[3] Ker J., Wang L., Rao J., Lim T. (2017). Deep learning applications in medical image analysis. *IEEE Access*.

[4] Jena M., Mishra S. P., Mishra D. (2018). A survey on applications of machine learning techniques for medical image segmentation. *International Journal of Engineering & Technology*.

[5] Niyas S., Pawan S. J., Anand Kumar M., Rajan J. (2022). Medical image segmentation with 3D convolutional neural networks: a survey. *Neurocomputing*.

[6] Dutta P., Upadhyay P., De M., Khalkar R. G. Medical image analysis using deep convolutional neural networks: CNN architectures and transfer learning.

[7] Jogin M., Mohana M. S. M., Divya G. D., Meghana R. K., Apoorva S. Feature extraction using convolution neural networks (CNN) and deep learning.

[8] Goodfellow I. J., Shlens J., Szegedy C. (2014). Explaining and harnessing adversarial examples.

[9] Hu Z., Tang J., Wang Z., Zhang K., Zhang L., Sun Q. (2018). Deep learning for image-based cancer detection and diagnosis − a survey. *Pattern Recognition*.

[10] Liu X., Song L., Liu S., Zhang Y. (2021). A review of deep-learning-Based medical image segmentation methods. *Sustainability*.

[11] Shin H. C., Roth H. R., Gao M. (2016). Deep convolutional neural networks for computer-aided detection: CNN architectures, dataset characteristics and transfer learning. *IEEE Transactions on Medical Imaging*.

[12] Kazeminia S., Baur C., Kuijper A. (2020). GANs for medical image analysis. *Artificial Intelligence in Medicine*.

[13] Fu Y., Lei Y., Wang T., Curran W. J., Liu T., Yang X. (2021). A review of deep learning based methods for medical image multi-organ segmentation. *Physica Medica*.

[14] Halalli B., Makandar A. (2018). Computer aided diagnosis-medical image analysis techniques. *Breast Imaging*.

[15] Badža M. M., Barjaktarović M. Č. (2020). Classification of brain tumors from MRI images using a convolutional neural network. *Applied Sciences*.

[16] Rachapudi V., Lavanya Devi G. (2020). Improved convolutional neural network based histopathological image classification. *Evolutionary Intelligence*.

[17] Shelhamer E., Long J., Darrell T. (2017). Fully convolutional networks for semantic segmentation. *IEEE Transactions on Pattern Analysis and Machine Intelligence*.

[18] Sun J., Peng Y., Guo Y., Li D. (2021). Segmentation of the multimodal brain tumor image used the multi-pathway architecture method based on 3D FCN. *Neurocomputing*.

[19] Ronneberger O., Fischer P., Brox T., Navab N., Hornegger J., Wells W., Frangi A. (2015). U-Net: convolutional networks for biomedical image segmentation. *Medical Image Computing and Computer-Assisted Intervention – MICCAI 2015. MICCAI 2015*.

[20] Dharwadkar N. V., Savvashe A. K. (2021). Right ventricle segmentation of magnetic resonance image using the modified convolutional neural network. *Arabian Journal for Science and Engineering*.

[21] Li C., Song X., Zhao H. (2021). An 8-layer residual U-net with deep supervision for segmentation of the left ventricle in cardiac CT angiography. *Computer Methods and Programs in Biomedicine*.

[22] Li C., Tan Y., Chen W. Attention unet++: a nested attention-aware U-net for liver CT image segmentation.

[23] Zhou Z., Rahman Siddiquee M. M., Tajbakhsh N., Liang J. (2018). Unet++: a nested u-net architecture for medical image segmentation. *Deep learning in medical image analysis and multimodal learning for clinical decision support*.

[24] Milletari F., Navab N., Ahmadi S. A. V-net: fully convolutional neural networks for volumetric medical image segmentation.

[25] Guan X., Yang G., Yang J., Xu X., Jiang W., Lai X. (2022). 3D AGSE-VNet: an automatic brain tumor MRI data segmentation framework. *BMC Medical Imaging*.

[26] He K., Gkioxari G., Dollar P., Girshick R. (2020). Mask R-CNN. *IEEE Transactions on Pattern Analysis and Machine Intelligence*.

[27] Dogan R. O., Dogan H., Bayrak C., Kayikcioglu T. (2021). A two-phase approach using mask R-CNN and 3D U-net for high-accuracy automatic segmentation of pancreas in CT imaging. *Computer Methods and Programs in Biomedicine*.

[28] Yamashita R., Nishio M., Do R. K. G., Togashi K. (2018). Convolutional neural networks: an overview and application in radiology. *Insights Into Imaging*.

[29] Lecun Y., Bottou L., Bengio Y., Haffner P. (1998). Gradient-based learning applied to document recognition. *Proceedings of the IEEE*.

[30] Krizhevsky A., Sutskever I., Hinton G. E. (2017). ImageNet classification with deep convolutional neural networks. *Communications of the ACM*.

[31] Simonyan K., Zisserman A. (2014). Very deep convolutional networks for large-scale image recognition.

[32] Szegedy C., Jia Y., Sermanet P. Going deeper with convolutions.

[33] He K., Zhang X., Ren S., Sun J. Deep residual learning for image recognition.

[34] Hazarika R. A., Abraham A., Kandar D., Maji A. K. (2021). An improved LeNet-deep neural network model for Alzheimer’s disease classification using brain magnetic resonance images. *IEEE Access*.

[35] Hosny K. M., Kassem M. A., Fouad M. M. (2020). Classification of skin lesions into seven classes using transfer learning with AlexNet. *Journal of Digital Imaging*.

[36] Dulf E. H., Bledea M., Mocan T., Mocan L. (2021). Automatic detection of colorectal polyps using transfer learning. *Sensors*.

[37] Szegedy C., Vanhoucke V., Ioffe S., Shlens J., Wojna Z. Rethinking the inception architecture for computer vision.

[38] Hameed Z., Zahia S., Garcia-Zapirain B., Javier Aguirre J., María Vanegas A. (2020). Breast cancer histopathology image classification using an ensemble of deep learning models. *Sensors*.

[39] Toğaçar M., Cömert Z., Ergen B. (2020). Classification of brain MRI using hyper column technique with convolutional neural network and feature selection method. *Expert Systems with Applications*.

[40] Eid M. M., Elawady Y. H. (2021). Efficient pneumonia detection for chest radiography using ResNet-Based SVM. *European Journal of Electrical Engineering and Computer Science*.

[41] Xiao Z., Liu B., Geng L., Zhang F., Liu Y. (2020). Segmentation of lung nodules using improved 3D-UNet neural network. *Symmetry*.

[42] Goyal M., Guo J., Hinojosa L., Hulsey K., Pedrosa I. (2022). Automated kidney segmentation by mask R-CNN in T2-weighted magnetic resonance imaging. *Medical Imaging 2022: Computer-Aided Diagnosis*.

[43] Goodfellow I., Pouget-Abadie J., Mirza M. (2020). Generative adversarial networks. *Communications of the ACM*.

[44] Cirillo M. D., Abramian D., Eklund A. (2021). Vox2Vox: 3D-GAN for brain tumour segmentation. *Brainlesion: Glioma, Multiple Sclerosis, Stroke and Traumatic Brain Injuries*.

[45] Wang W., Wang G., Wu X. (2021). Automatic segmentation of prostate magnetic resonance imaging using generative adversarial networks. *Clinical Imaging*.

[46] Wei X., Chen X., Lai C., Zhu Y., Yang H., Du Y. (2021). Automatic liver segmentation in CT images with enhanced GAN and mask region-Based CNN architectures. *BioMed Research International*.

[47] Ma J., Deng Y., Ma Z., Mao K., Chen Y. (2021). A liver segmentation method based on the fusion of VNet and WGAN. *Computational and Mathematical Methods in Medicine*.

[48] Arjovsky M., Chintala S., Bottou L. Wasserstein generative adversarial networks.

[49] Zhang J., Yu L., Chen D. (2021). Dense GAN and multi-layer attention based lesion segmentation method for COVID-19 CT images. *Biomedical Signal Processing and Control*.

[50] Waghmare V. K., Kolekar M. H. (2021). Brain tumor classification using deep learning. *Internet of Things for Healthcare Technologies*.

[51] Çinar A., Yildirim M. (2020). Detection of tumors on brain MRI images using the hybrid convolutional neural network architecture. *Medical Hypotheses*.

[52] Chen S., Zhang J., Wei X., Zhang Q. Alzheimer’s disease classification using structural MRI based on convolutional neural networks.

[53] Chouha V., Singh S. K., Khamparia A. (2020). A novel transfer learning based approach for pneumonia detection in chest X-ray images. *Applied Sciences*.

[54] Lin C. J., Li Y. C. (2020). Lung nodule classification using Taguchi-based convolutional neural networks for computer tomography images. *Electronics*.

[55] Abdel Rahman A. S., Belhaouari S. B., Bouzerdoum A., Baali H., Alam T., Eldaraa A. M. Breast mass tumor classification using deep learning.

[56] Al-Haija Q. A., Adebanjo A. Breast cancer diagnosis in histopathological images using ResNet-50 convolutional neural network.

[57] Saber A., Sakr M., Abo-Seida O. M., Keshk A., Chen H. (2021). A novel deep-learning model for automatic detection and classification of breast cancer using the transfer-learning technique. *IEEE Access*.

[58] Thurnhofer-Hemsi K., Domínguez E. (2020). A convolutional neural network framework for accurate skin cancer detection. *Neural Processing Letters*.

[59] Hosny K. M., Kassem M. A., Foaud M. M. (2020). Skin melanoma classification using ROI and data augmentation with deep convolutional neural networks. *Multimedia Tools and Applications*.

[60] Bakht A. B., Javed S., Dina R., Almarzouqi H., Khandoker A., Werghi N. Thyroid nodule cell classification in cytology images using transfer learning approach. *Proceedings of the 12th International Conference on Soft Computing and Pattern Recognition (SoCPaR 2020)*.

[61] Chen W., Gu Z., Liu Z. (2021). A new classification method in ultrasound images of benign and malignant thyroid nodules based on transfer learning and deep convolutional neural network. *Complexity*.

[62] Bhanothu Y., Kamalakannan A., Rajamanickam G. Detection and classification of brain tumor in MRI images using deep convolutional network.

[63] Salama W. M., Aly M. H. (2021). Deep learning in mammography images segmentation and classification: automated CNN approach. *Alexandria Engineering Journal*.

[64] Khouani A., El Habib Daho M., Mahmoudi S. A., Chikh M. A., Benzineb B. (2020). Automated recognition of white blood cells using deep learning. *Biomedical Engineering Letters*.

[65] Antoniou A., Storkey A., Edwards H. (2017). Data augmentation generative adversarial networks.

[66] Beynek B., Bora Ş., Evren V., Ugur A. (2021). Synthetic skin cancer image data generation using generative adversarial neural network. *International Journal of Multidisciplinary Studies and Innovative Technologies*.

[67] Ahmad B., Jun S., Palade V., You Q., Mao L., Zhongjie M. (2021). Improving skin cancer classification using heavy-tailed student T-distribution in generative adversarial networks (TED-GAN). *Diagnostics*.

[68] Yu H., Zhang X. (2020). Synthesis of prostate MR images for classification using capsule network-based GAN model. *Sensors*.

[69] Kaur S., Aggarwal H., Rani R. (2020). Diagnosis of Parkinson’s disease using deep CNN with transfer learning and data augmentation. *Multimedia Tools and Applications*.

[70] Mondal B., Das N., Santosh K. C., Nasipuri M. Improved skin disease classification using generative adversarial network.

[71] Pang T., Wong J. H. D., Ng W. L., Chan C. S. (2021). Semi-supervised GAN-based radiomics model for data augmentation in breast ultrasound mass classification. *Computer Methods and Programs in Biomedicine*.

[72] Ahmad B., Sun J., You Q., Palade V., Mao Z. (2022). Brain tumor classification using a combination of variational autoencoders and generative adversarial networks. *Biomedicine*.

[73] Li Y., Chen Y., Shi Y. (2020). Brain tumor segmentation using 3D generative adversarial networks. *International Journal of Pattern Recognition and Artificial Intelligence*.

[74] Negi A., Raj A. N. J., Nersisson R., Zhuang Z., Murugappan M. (2020). RDA-UNET-WGAN: an accurate breast ultrasound lesion segmentation using Wasserstein generative adversarial networks. *Arabian Journal for Science and Engineering*.

[75] Decourt C., Duong L. (2020). Semi-supervised generative adversarial networks for the segmentation of the left ventricle in pediatric MRI. *Computers in Biology and Medicine*.

[76] Chen L. C., Papandreou G., Kokkinos I., Murphy K., Yuille A. L. (2018). DeepLab: semantic image segmentation with deep convolutional nets, atrous convolution, and fully connected CRFs. *IEEE Transactions on Pattern Analysis and Machine Intelligence*.

[77] Lou Z., Huo W., Le K., Tian X. Whole heart auto segmentation of cardiac CT images using U-Net based GAN.

[78] Wu X., Bi L., Fulham M., Kim J. Unsupervised positron emission tomography tumor segmentation via GAN based adversarial auto-encoder.

[79] AlexSWong (2022). *AlexSWong/COVID-Net*.

[80] Wang L., Lin Z. Q., Wong A. (2020). COVID-Net: a tailored deep convolutional neural network design for detection of COVID-19 cases from chest X-ray images. *Scientific Reports*.

[81] Halabi S. S., Prevedello L. M., Kalpathy-Cramer J. (2019). The RSNA pediatric bone age machine learning challenge. *Radiology*.

[82] *RSNA Bone Age*.

[83] Luz E., Silva P., Silva R. (2022). Towards an effective and efficient deep learning model for COVID-19 patterns detection in X-ray images. *Research on Biomedical Engineering*.

[84] Punn N. S., Agarwal S. (2020). Automated diagnosis of COVID-19 with limited posteroanterior chest X-ray images using fine-tuned deep neural networks. *Applied Intelligence*.

[85] Waheed A., Goyal M., Gupta D., Khanna A., Al-Turjman F., Pinheiro P. R. (2020). Covid GAN: data augmentation using auxiliary classifier GAN for improved COVID-19 detection. *IEEE Access*.

[86] Oh Y., Park S., Ye J. C. (2020). Deep learning COVID-19 features on CXR using limited training data sets. *IEEE Transactions on Medical Imaging*.

[87] Wang N., Liu H., Xu C. Deep learning for the detection of COVID-19 using transfer learning and model integration.

[88] Li J., Zhang D., Liu Q., Bu R., Wei Q. COVID-GATNet: A deep learning framework for screening of COVID-19 from chest X-ray images.

[89] Ahsan M., Based M., Haider J., Kowalski M. (2021). COVID-19 detection from chest X-ray images using feature fusion and deep learning. *Sensors*.

[90] Al-Waisy A. S., Al-FahdawiS S., Mohammed M. A. (2023). COVID-CheXNet: hybrid deep learning framework for identifying COVID-19 virus in chest X-rays images. *Soft Computing*.

[91] Li X., Tan W., Liu P., Zhou Q., Yang J. (2021). Classification of COVID-19 chest CT images Based on ensemble deep learning. *Journal of Healthcare Engineering*.

[92] Pathak Y., Shukla P. K., Arya K. V. (2021). Deep bidirectional classification model for COVID-19 disease infected patients. *IEEE/ACM Transactions on Computational Biology and Bioinformatics*.

[93] Horry M. J., Chakraborty S., Paul M. (2020). COVID-19 detection through transfer learning using multimodal imaging data. *IEEE Access*.

[94] Iglovikov V. I., Rakhlin A., Kalinin A. A., Shvets A. A. (2018). Paediatric bone age assessment using deep convolutional neural networks. *Deep Learning in Medical Image Analysis and Multimodal Learning for Clinical Decision Support*.

[95] Pan X., Zhao Y., Chen H., Wei D., Zhao C., Wei Z. (2020). Fully automated bone age assessment on large-scale hand X-ray dataset. *International Journal of Biomedical Imaging*.

[96] Zulkifley M. A., Abdani S. R., Zulkifley N. H. (2020). Automated bone age assessment with image registration using hand X-ray images. *Applied Sciences*.

[97] Gao Y., Zhu T., Xu X. (2020). Bone age assessment based on deep convolution neural network incorporated with segmentation. *International Journal of Computer Assisted Radiology and Surgery*.

[98] Li S., Liu B., Li S., Zhu X., Yan Y., Zhang D. (2022). A deep learning-based computer-aided diagnosis method of X-ray images for bone age assessment. *Complex & Intelligent Systems*.

[99] Salim I., Hamza A. B. (2021). Ridge regression neural network for pediatric bone age assessment. *Multimedia Tools and Applications*.

